# Tumor Subtype-Specific Associations of Hormone-Related Reproductive Factors on Breast Cancer Survival

**DOI:** 10.1371/journal.pone.0123994

**Published:** 2015-04-14

**Authors:** Nan Song, Ji-Yeob Choi, Hyuna Sung, Sujee Jeon, Seokang Chung, Minkyo Song, Sue K. Park, Wonshik Han, Jong Won Lee, Mi Kyung Kim, Keun-Young Yoo, Sei-Hyun Ahn, Dong-Young Noh, Daehee Kang

**Affiliations:** 1 Cancer Research Institute, Seoul National University College of Medicine, Seoul, Korea; 2 Department of Biomedical Sciences, Seoul National University Graduate School, Seoul, Korea; 3 Department of Preventive Medicine, Seoul National University College of Medicine, Seoul, Korea; 4 Division of Epidemiology and Genetics, National Cancer Institute, Rockville, Maryland, United States of America; 5 Department of Surgery, Seoul National University College of Medicine, Seoul, Korea; 6 Department of Surgery, University of Ulsan College of Medicine and ASAN Medical Center, Seoul, Korea; 7 Division of Cancer Epidemiology and Management, National Cancer Center, Goyang-si, Gyeonggi-do, Korea; Shanghai Jiao Tong University School of Medicine, CHINA

## Abstract

**Purpose:**

It is inconclusive whether reproductive factors, which are known as risk factors of breast cancer, also influence survival. We investigated overall and subtype-specific associations between reproductive factors and breast cancer survival.

**Methods:**

Among 3,430 incident breast cancer patients who enrolled in the Seoul Breast Cancer Study, 269 patients (7.8%) died and 528 patients (15.4%) recurred. The overall and subtype-specific associations of reproductive factors including age at menarche and menopause, duration of estrogen exposure, menstrual cycle, parity, age at first full-term pregnancy, number of children, age at last birth, time since the last birth, and duration of breastfeeding, on overall and disease-free survival (OS and DFS) were estimated by hazard ratios (HRs) and 95% confidence intervals (95% CIs) using a multivariate Cox proportional hazard model.

**Results:**

An older age at menarche (HR for OS=1.10, 95% CI=1.03-1.19), a greater number of children (≥4 vs. 2, HR for DFS=1.58, 95% CI=1.11-2.26), and a shorter time since last birth (<5 vs. ≥20 years, HR for DFS=1.67, 95% CI=1.07-2.62) were associated with worse survival while longer duration of estrogen exposure with better survival (HR for DFS=0.97, 95% CI=0.96-0.99). In the stratified analyses by subtypes, those associations were more pronounced among women with hormone receptor and human epidermal growth factor 2 positive (HR+ HER2+) tumors.

**Conclusions:**

It is suggested that reproductive factors, specifically age at menarche, number of children, time since last birth, and duration of estrogen exposure, could influence breast tumor progression, especially in the HR+ HER2+ subtype.

## Introduction

Reproductive factors associated with prolonged exposure of endogenous estrogen exposure affect the pathogenesis of breast cancer[1]. However, their effects on overall breast cancer survival have been inconsistent in previous studies on age at menarche[2,3], age at first full-term pregnancy (FFTP)[4–8], status[5,9–14] or number[5,7,10,15–19] of parity, and number of years since last birth[5,7,13–15,18–27].

Breast cancer exhibits heterogeneous clinical behaviors according to intrinsic tumor subtypes which are defined based on their expression of ER, PR, and human epidermal growth factor receptor 2 (HER2)[28]. Reproductive factors are not only associated with intrinsic tumor subtypes[29] but also with diverse clinicopathological characteristics which include tumor stage, grade, histologic subtype and estrogen and progesterone receptor (ER and PR) status, and the differences in the associations with breast cancer survival could be due to these reproductive factors [30,31].

Thus, the aim of this study was to investigate associations between reproductive factors and breast cancer survival according to diverse clinicopathological subgroups including intrinsic tumor subtypes.

## Materials and Methods

### Study population

This is a follow-up study that included eligible patients previously enrolled in the Seoul Breast Cancer Study (SEBCS), a multicenter-based case-control study on breast cancer described elsewhere[32,33]. The patients with histologically confirmed incident breast cancer at department of surgery of Seoul National University Hospital (SNUH) or ASAN Medical Center (AMC) were recruited and enrolled in the SEBCS between 2001 and 2007. All patients had breast cancer surgery. As university-affiliated tertiary general hospitals, the two recruiting centers covered 19.9% of the surgery for Korean breast cancer patients in 2010. Among 4,040 registered subjects, 351 patients with a previous history of cancer and/or benign breast disease were excluded from the study leaving a remaining 3,689 patients. Among remaining subjects, 53 breast cancer patients with metastasis were also excluded. Additionally, 175 patients with missing follow-up information and 31 patients with a follow-up period of less than 90 days were excluded. Thus, a total of 3,430 incident breast cancer patients were included in the analysis.

### Data collection

From all the participants in the study, detailed information on demographic factors, family history of diseases, reproductive factors, and lifestyle factors at enrollment were collected by trained interviewers with face-to-face surveys using structured questionnaires. The clinicopathological information for tumor stage and grade, estrogen and progesterone receptor (ER and PR), human epidermal growth factor receptor 2 (HER2), and treatment were obtained by reviewing the medical records of the patients. However, the substantial treatment information was missed in medical records; The proportions of missing data in adjuvant chemotherapy, hormone therapy, and radiotherapy were 15.0%, 22.3%, and 20.6%, respectively. All the study participants provided written informed consent. This study was approved by the Committee on Human Research at Seoul National University Hospital (IRB No. H-0503-144-004).

### Follow-up

The vital status of the patients, specifically breast cancer recurrence and all-cause mortality, was obtained through a retrospective review of the medical records of the patients up until 2011. In addition, death certificates from the National Statistical Office in Korea were used to confirm deaths. The breast cancer patients known to be alive at the date of last follow-up without a vital status were censored. Survival time was calculated from the date of the breast cancer surgery to loco-regional recurrence, first distant metastasis, 2^nd^ primary cancer, or death from any cause for disease-free survival (DFS), and overall survival (OS) was calculated from the date of the breast cancer surgery to death, or if the patients were censored, OS was calculated from the date of the breast cancer surgery to the last follow-up.

### Variables

Baseline characteristics consisted of general demographic and epidemiological factors, including age at diagnosis (<40, 40–49, 50–59, and ≥60 years), education (less than high school, high school, and college or higher), family history of breast cancer (no or yes), body mass index (BMI, <18.5, 18.5–22.9, 23.0–24.9, and ≥25.0 kg/m^2^), and menopausal status (pre- and postmenopausal) and clinicopathological factors, such as tumor size (≤2, 2.1–5.0, and >5.0 cm) nodal status (negative or positive; 1–3, 4–9, and ≥10 nodes), TNM stage (0, I, II, and III) according to the 7th edition of the American Joint Committee on Cancer (AJCC), histological and nuclear grade (1, 2, and 3), ER, PR, HR, and HER2 status (negative and positive), intrinsic subtypes (HR+ HER2-, HR+ HER2+, HR- HER2+, and HR- HER2-), adjuvant chemotherapy, hormone therapy, and radiotherapy (yes or no).

Reproductive factors were the main exposure variables and included age at menarche (≤13, 14–15, and ≥16 years, as the tertiles), age at menopause among postmenopausal women (≤47, 47–51, and ≥52 years, as the tertiles), menstrual cycle indicated as the answer to the questionnaire (Have you had a regular menstruation? yes (menstrual cycle ranging from 28 to 35 days) or no), parity (parous and nulliparous), and duration of lifetime endogenous estrogen exposure (≤27, 28–33, and ≥34 years, as the tertiles) or before FFTP (≤9, 10–13, and ≥14 years, as the tertiles) for all women and age at FFTP (≤24, 25–27, and ≥28 years, as the tertiles), number of children (1, 2, 3, and ≥4), age at last birth (≤28, 29–31, and ≥32 years, as the tertiles), number of years since last birth (<5, 5–9, 10–14, 15–19, ≥20 years), and duration of breastfeeding (≤9, 10–13, and ≥14 months, as the tertiles) for parous women. The duration of lifetime endogenous estrogen exposure was calculated by subtracting the age at menarche from the age at diagnosis for premenopausal women and from the age at menopause for postmenopausal women. The duration of endogenous estrogen exposure before FFTP was defined as the period between menarche and FFTP for parous women and the period between menarche and diagnosis or menopause for pre- and postmenopausal nulliparous women, respectively, as previously described[33]. The reference groups were defined as the first category of each variable and the greater proportion in each variable including age and BMI for the statistical stability. Outcome variables were considered as all-cause deaths for OS and recurrences and all-cause deaths for DFS.

### Statistical analyses

To estimate the hazard ratios (HRs) and 95% confidence intervals (CIs) of the associations between the characteristics and breast cancer recurrences or deaths, multivariate Cox proportional hazard model was used adjusting for age (continuous), recruiting centers (SNUH and AMC), menopausal status (pre- and postmenopausal), TNM stage (0, I, II, and III), and intrinsic subtypes (HR+ HER2-, HR+ HER2+, HR- HER2+, and HR- HER2-). The baseline characteristics were evaluated as potential confounders, but adjusting factors were selected considering the statistical significance (*P*<0.05) of the association with both the OS and DFS of breast cancer. Since the analyses were not influenced by histological and nuclear grade, those factors were not considered as adjusting factors. The reproductive factors which had significant effects on survival had been additionally adjusted for the analyses as a fully adjusted model, but the results were similar to the reduced model which was adjusted age, recruiting centers, menopausal status, TNM stage, and intrinsic subtypes (data not shown). Thus, the reduced model was shown in the results. The analyses that included the variable for duration of breastfeeding were additionally adjusted for the number of children because of the statistically significant association between the two factors. For reproductive variables, the proportional hazard assumptions were satisfied based on *p*-values from Kolmogorov-type supremum tests.

For statistically significant reproductive factors on survival, such as age at menarche (per 1 year increase), duration of endogenous estrogen exposure (per 1 year increase), number of children (≥4 vs. 2), and number of years since last birth (<5 vs. ≥20 years), additional stratified analyses were conducted by diverse subgroups including age, menopausal status, histological and nuclear grade, TNM stage, ER, PR, HR, and HER2 status, and intrinsic subtypes, and the heterogeneity of the associations on breast cancer survival between the strata was tested with Cochran’s Q and I^2^ statistics across each category. Because interaction between the number of children and number of years since last birth was not statistically significant, the interaction term was not included in the survival model. Because of multiple testing, the false discovery rate (FDR) based on *p*-values from the analyses and Bonferroni correction (*p*-value = 0.05/11 = 0.005) were estimated. As a sensitivity analysis, the survival analysis was additionally conducted in 3,073 invasive breast cancer patients after excluding with TNM stage 0. The baseline characteristics of remained and excluded subjects because of follow-up loss were compared by chi-square test. All statistical analyses were considered as two-sided and performed by SAS 9.3 and STATA 12.1.

## Results

The baseline characteristics and those influencing the survival of breast cancer are presented in Table 1. Among the 3,430 incident breast cancer patients, there were 269 (7.8%) events for OS and 528 (15.4%) events for DFS during the follow-up period (median_OS_ = 4.42, range = 0.25–9.59 years and median_DFS_ = 4.28, range = 0.25–9.67 years). As shown in Table 1, the OS and DFS were worse for women with the following characteristics: younger age than 40 years, aged 60 or older, postmenopausal, larger tumor size and positive lymph nodes, advanced TNM stage and histological and nuclear grade, ER-, PR-, and HR-negative, and HR+ HER2+, HR- HER2+, and HR- HER2- subtypes compared to the HR+ HER2- subtype (*P*<0.05). The other characteristics including education, family history of breast cancer, BMI, HER2, adjuvant chemotherapy, hormone therapy, and radiotherapy were not associated with breast cancer survival (*P*>0.05). Although 175 subjects (4.8% of 3,636 subjects for the survival analysis) were excluded in the analysis because of follow-up loss through the medical chart review, there were not statistically significant differences of characteristics including age, family history of breast cancer, BMI, menopausal status, TNM stage, and intrinsic subtypes at the diagnosis between remained and excluded subjects because of follow-up loss (S1 Table).

**Table 1:** Baseline characteristics and prognosis of breast cancer patients in the Seoul Breast Cancer Study (SEBCS).

Characteristics	All		Event_OS_		HR^a^	(95% CI)	Event_DFS_		HR^a^	(95% CI)
	(N = 3,430)		(N = 269, 7.8%)				(N = 528, 15.4%)			
	N	(%)	N	(%)			N	(%)		
Age at diagnosis, years										
<40	674	(19.7)	73	(27.1)	1.39	(1.02–1.90)	134	(25.4)	1.46	(1.17–1.83)
40–49	1,443	(42.1)	92	(34.2)	1.00	ref.	180	(34.1)	1.00	ref.
50–59	871	(25.4)	58	(21.6)	0.94	(0.67–1.32)	128	(24.2)	0.93	(0.93–1.47)
≥60	442	(12.9)	46	(17.1)	1.60	(1.12–2.29)	86	(16.3)	1.22	(1.22–2.04)
Education										
less than high school	937	(27.3)	86	(32.0)	1.00	ref.	158	(29.9)	1.00	ref.
high school	1,344	(39.2)	99	(36.8)	0.87	(0.64–1.20)	201	(38.1)	0.97	(0.78–1.22)
college or higher	1,124	(32.8)	81	(30.1)	0.84	(0.60–1.19)	165	(31.3)	0.99	(0.78–1.27)
Family history of breast cancer										
no	3,266	(95.2)	261	(97.0)	1.00	ref.	502	(95.1)	1.00	ref.
yes	162	(4.7)	8	(3.0)	0.58	(0.27–1.23)	26	(4.9)	1.01	(0.67–1.51)
BMI, kg/m^2^										
<18.5	119	(3.5)	13	(4.8)	1.26	(0.71–2.25)	23	(4.4)	1.24	(0.81–1.90)
18.5–22.9	1,675	(48.8)	126	(46.8)	1.00	ref.	252	(47.7)	1.00	ref.
23.0–24.9	819	(23.9)	59	(21.9)	0.87	(0.63–1.19)	112	(21.2)	0.82	(0.66–1.03)
≥25.0	790	(23.0)	67	(24.9)	1.03	(0.76–1.39)	136	(25.8)	1.02	(0.82–1.27)
Menopausal status										
premenopausal	2,145	(62.5)	159	(59.1)	1.00	ref.	305	(57.8)	1.00	ref.
postmenopausal	1,259	(36.7)	107	(39.8)	1.13	(0.79–1.62)	219	(41.5)	1.30	(1.01–1.68)
Tumor size, cm										
≤2.0	1,894	(55.2)	61	(22.7)	1.00	ref.	165	(31.3)	1.00	ref.
2.1–5.0	1,308	(38.1)	152	(56.5)	2.14	(1.56–2.94)	273	(51.7)	1.66	(1.35–2.04)
>5.0	194	(5.7)	47	(17.5)	3.61	(2.36–5.52)	79	(15.0)	2.86	(2.12–3.86)
Nodal status, n										
negative	2,136	(62.3)	79	(29.4)	1.00	ref.	197	(37.3)	1.00	ref.
positive, 1–3	878	(25.6)	103	(38.3)	2.22	(1.62–3.03)	184	(34.9)	1.86	(1.50–2.31)
positive, 4–9	244	(7.1)	41	(15.2)	2.99	(1.98–4.53)	74	(14.0)	2.54	(1.89–3.40)
positive, ≥10	172	(5.0)	46	(17.1)	5.22	(3.45–7.89)	73	(13.8)	3.99	(2.95–5.39)
TNM stage										
0-I	1,503	(43.8)	33	(12.3)	1.00	ref.	110	(20.8)	1.00	ref.
II	1,441	(42.0)	132	(49.1)	3.43	(2.28–5.17)	245	(46.4)	2.18	(1.74–2.73)
III	485	(14.1)	104	(38.7)	9.63	(6.29–14.75)	173	(32.8)	5.56	(4.36–7.10)
Histological grade										
1	215	(6.3)	4	(1.5)	1.00	ref.	12	(2.3)	1.00	ref.
2	1,397	(40.7)	70	(26.0)	1.56	(0.56–4.29)	162	(30.7)	1.48	(0.82–2.66)
3	1,185	(34.6)	155	(57.6)	2.75	(1.00–7.58)	282	(53.4)	2.41	(1.33–4.35)
unknown	633	(18.4)	40	(14.9)			72	(13.6)		
Nuclear grade										
1	213	(6.2)	17	(6.3)	1.00	ref.	33	(6.3)	1.00	ref.
2	1,262	(36.8)	50	(18.6)	0.71	(0.40–1.27)	137	(26.0)	0.86	(0.58–1.28)
3	974	(28.4)	98	(36.4)	1.25	(0.73–2.15)	203	(38.5)	1.34	(0.91–1.96)
unknown	984	(28.7)	104	(38.7)			155	(29.4)		
Estrogen receptor (ER)										
positive	2,101	(61.3)	107	(39.8)	1.00	ref.	259	(49.1)	1.00	ref.
negative	1,250	(36.4)	158	(58.7)	1.91	(1.40–2.59)	262	(49.6)	1.42	(1.15–1.75)
unknown	79	(2.3)	4	(1.5)			7	(1.3)		
Progesterone receptor (PR)										
positive	1,783	(52.0)	77	(28.6)	1.00	ref.	196	(37.1)	1.00	ref.
negative	1,564	(45.6)	187	(69.5)	1.82	(1.31–2.52)	324	(61.4)	1.55	(1.25–1.93)
unknown	83	(2.4)	5	(1.9)			8	(1.5)		
Hormone receptor (HR)										
positive (ER+ or PR+)	2,395	(69.8)	128	(47.6)	1.00	ref.	297	(56.3)	1.00	ref.
negative (ER- and PR-)	954	(27.8)	136	(50.6)	2.42	(1.85–3.18)	223	(42.2)	1.86	(1.54–2.26)
unknown	81	(2.4)	5	(1.9)			8	(1.5)		
HER2										
negative	2,093	(61.0)	147	(54.7)	1.00	ref.	298	(56.4)	1.00	ref.
positive	795	(23.2)	85	(31.6)	1.12	(0.84–1.49)	156	(29.6)	1.16	(0.94–1.42)
unknown	542	(15.8)	37	(13.8)			74	(14.0)		
Intrinsic subtypes										
HR+ HER2-	1,615	(47.1)	74	(27.5)	1.00	ref.	180	(34.1)	1.00	ref.
HR+ HER2+	398	(11.6)	30	(11.2)	1.44	(0.93–2.21)	64	(12.1)	1.38	(1.04–1.85)
HR- HER2+	395	(11.5)	55	(20.5)	2.61	(1.83–3.73)	92	(17.4)	2.06	(1.60–2.67)
HR- HER2-	473	(13.8)	72	(26.8)	2.77	(1.99–3.86)	117	(22.2)	2.11	(1.67–2.67)
unknown	549	(16.0)	38	(14.1)			75	(14.2)		
Adjuvant chemotherapy										
yes	2,334	(68.1)	236	(87.7)	1.00	ref.	439	(83.1)	1.00	ref.
no	582	(17.0)	15	(5.6)	1.02	(0.54–1.91)	41	(7.8)	0.90	(0.61–1.33)
unknown	514	(15.0)	18	(6.7)			48	(9.1)		
Adjuvant hormone therapy										
yes	2,016	(58.8)	97	(36.1)	1.00	ref.	2,019	(58.8)	1.00	ref.
no	649	(18.9)	71	(26.4)	1.32	(0.75–2.31)	649	(18.9)	1.21	(0.83–1.77)
unknown	765	(22.3)	101	(37.6)			765	(22.3)		
Adjuvant radiotherapy										
yes	1,874	(54.6)	159	(59.1)	1.00	ref.	302	(57.2)	1.00	ref.
no	849	(24.8)	64	(23.8)	1.02	(0.72–1.46)	139	(26.3)	1.30	(1.01–1.68)
unknown	707	(20.6)	46	(17.1)			87	(16.5)		

Abbreviations: Overall survival (OS); disease-free survival (DFS); hazard ratio (HR); confidence interval (CI).

^a^Adjusted for age, recruiting centers, TNM stage, and intrinsic subtypes.

**Fig 1:**
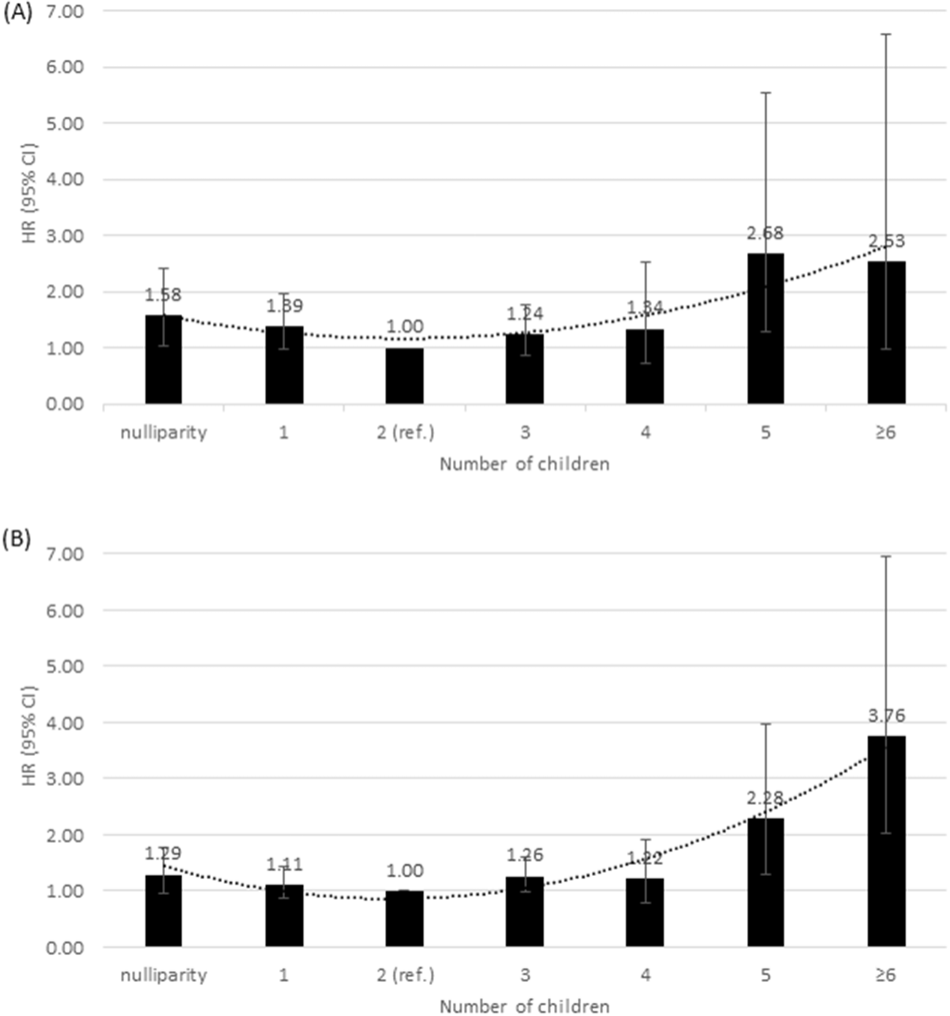
Hazard ratios (HRs) with 95% confidence intervals (CIs) for number of children and prognosis of breast cancer. (A) overall survival (OS). (B) disease-free survival (DFS). Estimates were adjusted for age, recruiting centers, TNM stage, and intrinsic subtypes.

Table 2 presents the results of the multivariate analyses on the associations between hormone-related reproductive factors and breast cancer survival. The OS was worse in women with an older age per 1 year at menarche. Similarly, a longer duration of endogenous estrogen exposure per 1 year was associated with a better DFS and OS with statistical significance by Bonferroni correction (*P*_DFS_ = 0.0006 and *P*_OS_ = 0.005). Among parous women, a dual-effect from the number of children on breast cancer survival was observed with the worse OS in women with 1 birth and 4 or worse DFS in women with more births compared to those with 2 births. A dual-effect from the number of children on breast cancer survival was also observed when investigated in more detail (Fig 1). Compared to women with 20 or more years since last birth, women with less than 5 years since last birth had a greater increased risk of recurrence and death. The age at menopause, menstrual cycle, parity, age at FFTP and last birth, duration of breastfeeding, and duration of endogenous estrogen exposure before FFTP were not associated with breast cancer survival (*P*>0.05). When the survival analysis was conducted stratified by TNM stage the association of age at menarche, duration of endogenous estrogen exposure, number of children, and number of years since last birth on survival were attenuated in early stage (TNM stage 0-I) patients in S1 File. Similarly, those associations tended to be attenuated among patients with treatments (S1 File).

**Table 2:** Reproductive factors and prognosis of breast cancer patients in the Seoul Breast Cancer Study (SEBCS).

Characteristics	All		Event_OS_		HR^a^	(95% CI)	Event_DFS_		HR^a^	(95% CI)
	(N = 3,430)		(N = 269, 7.8%)				(N = 528, 15.4%)			
	N	(%)	N	(%)			N	(%)		
Age at menarche, years										
≤13	711	(20.7)	39	(14.5)	0.80	(0.56–1.15)	95	(18.0)	0.90	(0.71–1.14)
14–15	1,616	(47.1)	123	(45.7)	1.00	ref.	252	(47.7)	1.00	ref.
≥16	1,064	(31.0)	102	(37.9)	1.22	(0.92–1.62)	173	(32.8)	0.95	(0.78–1.17)
per 1 year increase					1.10	(1.03–1.19)			1.02	(0.96–1.07)
Age at menopause among postmenopausal women, years										
≤47	365	(28.3)	22	(20.0)	0.97	(0.56–1.66)	50	(22.4)	0.91	(0.63–1.31)
47–51	437	(33.9)	36	(32.7)	1.00	ref.	72	(32.3)	1.00	ref.
≥52	390	(30.3)	43	(39.1)	1.25	(0.79–1.98)	82	(36.8)	1.15	(0.83–1.58)
per 1 year increase					1.02	(0.98–1.06)			1.02	(0.99–1.05)
Duration of endogenous estrogen exposure, years										
≤27	1,086	(31.6)	96	(35.7)	1.00	ref.	182	(34.5)	1.00	ref.
28–33	1,200	(35.0)	87	(32.3)	0.84	(0.60–1.17)	172	(32.6)	0.86	(0.67–1.09)
≥34	1,030	(30.0)	74	(27.5)	0.79	(0.52–1.20)	151	(28.6)	0.85	(0.64–1.14)
per 1 year increase					0.96	(0.94–0.98)			0.97	(0.96–0.99)
Duration of endogenous estrogen exposure before first full-term pregnancy (FFTP), years										
≤9	1,109	(32.3)	100	(37.2)	1.00	ref.	193	(36.6)	1.00	ref.
10–13	1,215	(35.4)	89	(33.1)	0.76	(0.56–1.02)	169	(32.0)	0.81	(0.65–1.00)
≥14	1,051	(30.6)	72	(26.8)	0.78	(0.56–1.09)	155	(29.4)	0.94	(0.74–1.18)
per 1 year increase					0.98	(0.95–1.00)			0.99	(0.98–1.01)
Menstrual cycle										
regular	2,294	(66.8)	197	(73.2)	1.00	ref.	366	(69.3)	1.00	ref.
irregular	490	(14.3)	55	(20.5)	1.01	(0.72–1.40)	94	(17.8)	0.98	(0.77–1.26)
Parity										
parous	3,134	(91.3)	241	(89.6)	1.00	ref.	480	(90.9)	1.00	ref.
nulliparous	297	(8.7)	28	(10.4)	1.46	(0.97–2.18)	48	(9.1)	1.25	(0.92–1.69)
Age at first full-term pregnancy (FFTP) among parous women, years										
≤24	1,068	(34.1)	83	(34.4)	1.00	ref.	176	(36.7)	1.00	ref.
25–27	1,144	(37.3)	90	(37.3)	1.03	(0.75–1.40)	164	(34.2)	0.91	(0.73–1.13)
≥28	905	(28.9)	67	(27.8)	1.01	(0.72–1.42)	138	(28.8)	1.03	(0.81–1.30)
per 1 year increase					0.99	(0.95–1.03)			1.00	(0.97–1.02)
Number of children among parous women										
1	493	(15.7)	45	(18.7)	1.41	(1.00–1.99)	76	(15.8)	1.13	(0.87–1.50)
2	1,825	(58.2)	122	(50.6)	1.00	ref.	250	(52.1)	1.00	ref.
3	533	(17.0)	46	(19.1)	1.18	(0.83–1.68)	95	(19.8)	1.21	(0.95–1.55)
≥4	228	(7.3)	26	(10.8)	1.56	(0.95–2.58)	52	(10.8)	1.58	(1.11–2.26)
Age at last birth among parous women, years										
≤28	962	(30.7)	93	(39.0)	1.00	ref.	159	(33.1)	1.00	ref.
29–31	851	(27.2)	62	(25.7)	0.75	(0.54–1.05)	133	(27.7)	0.98	(0.77–1.23)
≥32	895	(28.6)	79	(32.8)	0.98	(0.72–1.33)	144	(30.0)	1.05	(0.84–1.32)
per 1 year increase					1.00	(0.97–1.03)			1.00	(0.98–1.03)
Number of years since last birth among parous women, years										
≥20	1,232	(45.3)	116	(49.4)	1.00	ref.	218	(49.9)	1.00	ref.
15–19	455	(16.7)	31	(13.2)	0.95	(0.60–1.52)	57	(13.0)	0.81	(0.58–1.14)
10–14	445	(16.4)	28	(11.9)	0.91	(0.54–1.53)	50	(11.4)	0.76	(0.52–1.10)
5–9	349	(12.8)	28	(11.9)	1.16	(0.66–2.04)	58	(13.3)	1.13	(0.76–1.68)
<5	237	(8.7)	32	(13.6)	1.99	(1.07–3.70)	54	(12.4)	1.67	(1.07–2.62)
per 1 year decrease					1.00	(0.97–1.03)			1.00	(0.98–1.02)
Duration of breastfeeding among parous women^b^, months										
never	667	(21.3)	38	(15.8)	1.00	ref.	92	(19.2)	1.00	ref.
≤12	860	(27.4)	74	(30.7)	1.24	(0.82–1.86)	122	(25.4)	0.89	(0.67–1.19)
13–24	598	(19.1)	55	(22.8)	1.41	(0.90–2.20)	96	(20.0)	1.01	(0.74–1.37)
>24	626	(20.0)	70	(29.1)	1.60	(0.96–2.66)	126	(26.3)	1.09	(0.76–1.57)
per 1 month increase					1.01	(1.00–1.01)			1.00	(1.00–1.01)

Abbreviations: Overall survival (OS); disease-free survival (DFS); hazard ratio (HR); confidence interval (CI).

^a^Adjusted for age, recruiting centers, menopausal status, TNM stage, and intrinsic subtypes.

^b^Additionally adjusted for number of children.

**Fig 2:**
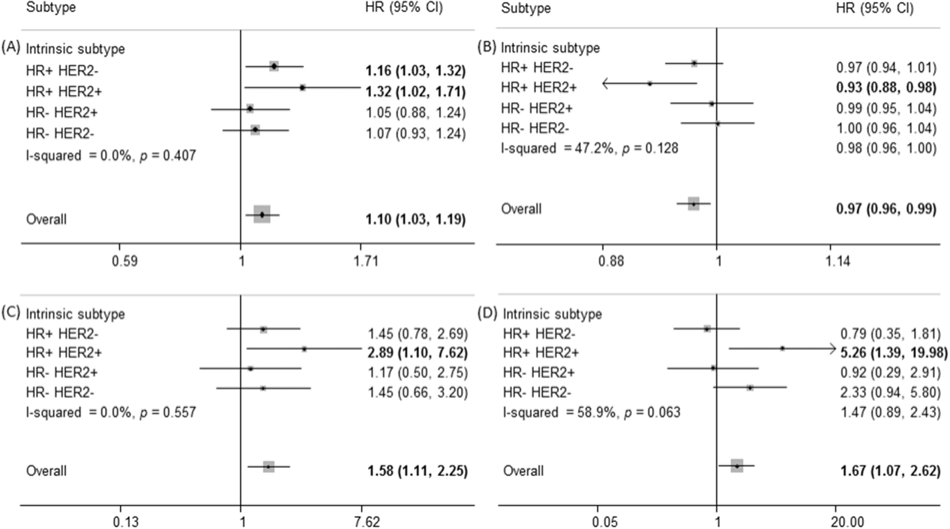
Hazard ratios (HRs) with 95% confidence intervals (CIs) for (A) age at menarche (per 1 year increase) on overall survival (OS) and (B) duration of endogenous estrogen exposure (per 1 year increase), (C) number of children ≥4 vs. 2, and (D) number of years since last birth <5 vs. ≥20 years on disease-free survival (DFS) of breast cancer by intrinsic subtypes. ^a^I-squared describes the percentage of the variability in effect estimates that is due to heterogeneity rather than sampling error.

We found no overall significant heterogeneity across clinicopathological subgroups; however, of note is the results of the analyses showing that the influences of the age at menarche, duration of endogenous estrogen exposure, number of children, and number of years since last birth on breast cancer survival seemed to be more pronounced in HR+ HER2+ tumors (Fig 2). A smaller number of years since last birth less than 5 years was also associated with postmenopausal breast cancer survival and there was significant heterogeneity between menopausal status (*P* = 0.005, S1 File). There were no other statistically significant differences across age, tumor grade and stage, and ER, PR, and HER2 status (*P*>0.05, S1 File). The analysis without TNM stage 0 showed similar associations of reproductive factors on overall and subtype-specific prognosis (S2 Table and S2 File).

## Discussion

This study showed that a prolonged duration of endogenous estrogen exposure tended to have better breast cancer survival. Moreover, parity-related factors including a parity of 4 or more and a smaller number of years since last birth (less than 5 years) were related to a poorer survival for breast cancer. The effects of those reproductive factors on breast cancer survival seemed to be more pronounced in women with HR+ HER2+ tumors.

Although related factors to prolonged estrogen exposure including early menarche and late menopause are well-known risk factors of breast cancer, there is insufficient evidence showing the effect of estrogen on cancer survival[2,3]. In the stratified analysis by diverse tumor subtypes in this study, longer estrogen exposure showed better survival in ER+, PR+, and HR+ HER2+ tumors, but not in HER2+ tumors (Fig 2C and Fig C in S1 File). The HR+ tumors, which consisted of ER+ and/or PR+, have a better cancer survival than that of the HR- tumors[34], and have a greater tendency for a longer duration of estrogen exposure[33]. Thus, the better cancer survival in women with prolonged estrogen exposure could be different based on the distribution of the tumor subtypes.

Similar results on poorer breast cancer survival related to high parity (3 or more births) versus nulliparity or 1–2 births have also been reported in several previous studies[7,10,15–19]. The dual effect of parity in women for which 2 births had a higher survival than less than 2 births or more than 2 births was also consistently observed in a general population as well as in breast cancer patients[5,35,36].

Many studies have shown a poorer survival in women who have gave birth within 5 years of the diagnosis of breast cancer[5,7,13–15,18–27]. This study also showed that women who gave birth within 5 years of the diagnosis of breast cancer were associated with a poorer survival than in women who gave birth 20 or more years before the diagnosis. The transient adverse effect of the comparably short duration on survival had been considered to be related to a younger age, an advanced clinicopathological stage or grade of disease, axillary nodes, and ER or PR- tumors in several studies[7,13,22,23,26]. Although it was considered that women who had last birth within 5 years were younger, the survival was poorer in the postmenopausal women (Fig D in S1 File). The women with a recent birth and menopause could have declined ovarian function and subsequent health problems, such as osteoporosis and cardiovascular disease suggesting the poorer survival[37].

During pregnancy and lactation, it is expected that women would experience transiently different gestational hormonal environments, such as increased estrogen, progesterone, prolactin, and growth hormone concentrations stimulating breast tumor cells[38]. In particular, prolactin contributes to metastasis and worse survival preventing apoptosis[39] and promoting cell motility[40] and angiogenesis[41] in breast cancer. Among peri- or postmenopausal women, higher expressed prolactin levels tended to be associated with increasing parity and also worse survival[42]. The poorer survival of women with higher parity and a recent birth of a child could also be explained by related health problems. Women with higher parity were considered to have the physical, physiological, and financial stresses of a larger family unit[35,36], and women with a recent birth of a child and menopause could have reduced ovarian function and subsequent health problems[37].

During pregnancy, the interplay between estrogen, progesterone, growth factor, and prolactin promotes mammary development by activating the expression of each receptor[43]. Prolactin exhibits luminal features such as higher levels of *Esr1*, *FoxA1*, and *Xbp1* mRNA transcripts[44]. In addition, breast tumors with HER2 overexpression had higher proliferation and metastasis with the presence of prolactin stimulating tyrosine phosphorylation of HER2 and activating mitogen-activated protein kinase (MAPK) signaling[45]. Among advanced breast cancer patients, hyperprolactinemia with HER2 expression was associated with an unfavorable prognosis[46]. Therefore, the parity-related effects on breast cancer survival could be profound in HR+ HER2+ tumors.

The strength of the current study is that it analyzed the profiles of reproductive factors by tumor subtypes based on the ER, PR, and HER2 status. In particular, the effect of the duration of estrogen exposure on breast cancer survival has not been estimated in previous studies. Through stratified analyses, whether different subtypes have a role as an effect modifier was evaluated.

The one limitation of this study was that patients were recruited from two centers (SNUH and AMC) and could have different clinicopathological characteristics that affect breast cancer survival. To control for relevant potential bias, recruiting centers as well as age, menopausal status, TNM stage, and intrinsic subtypes were adjusted for in all the analyses. Although 4.8% (175 of 3,636) subjects were lost to follow-up, the epidemiological and clinicopathological characteristics at the diagnosis were not different between remained and excluded subjects because of follow-up, which may not impact results of DFS. Contrary the common thought, the potential risk factors of breast cancer including prolonged estrogen exposure and more number of children were associated with the better prognosis. The similar results were observed as thrombophilia paradox[47], aspirin paradox[48], obesity paradox[49], and risk factor paradox in rheumatic disease[50]. This index event bias could be induced by selection of subjects based on the occurrence of the index event like breast cancer[51]. Contrary to previous meta analyses[52], our results did not show an association of BMI to breast cancer survival. However, the association was attenuated in Asians[53,54] and not statistically significant in Koreans[55]. Furthermore, we did not consider potentially changeable factors, such as BMI, physical activity, alcohol intake, smoking, and diet because the relevant data were collected at enrollment only. Other information on oral contraceptive use, hormone replacement therapy (HRT), quality of life, delayed treatment, and complications after breast cancer diagnosis was not collected and could not be considered in the analyses. The treatments including adjuvant chemotherapy, hormone therapy, and radiotherapy were also not controlled because of substantial missing data. When the analysis was focused among patients with those treatments, the associations tended to be attenuated because of the smaller sample size (S1 Table). Finally, a false discovery rate (FDR) control was not applied because the statistical power of the analysis was limited due to further stratification by clinicopathological subgroups. Among significant associations of reproductive factors on survival, the value of prolonged endogenous estrogen exposure was statistically significant even if Bonferroni corrected *p*-value was applied.

In conclusion, reproductive factors, specifically the number of children, number of years since last birth, and duration of endogenous estrogen exposure were associated with breast cancer survival, and the associations were more pronounced in HR+ HER2+ tumors, suggesting a hormone-related biological behavior in breast tumor progression.

## Supporting Information

S1 File Hazard ratios (HRs) with 95% confidence intervals (CIs) for (A) age at menarche (per 1 year increase) on overall survival (OS) and (B) duration of endogenous estrogen exposure (per 1 year increase), (C) number of children ≥4 vs. 2, and (D) number of years since last birth <5 vs. ≥20 years^a^ on disease-free survival (DFS) of breast cancer by clinicopathological subgroups. ^a^The sample size was not sufficient to analyze the stratified analysis by age categories.
(PPTX)

S2 File Hazard ratios (HRs) with 95% confidence intervals (CIs) for (A) age at menarche (per 1 year increase) on overall survival (OS) and (B) duration of endogenous estrogen exposure (per 1 year increase), (C) number of children ≥4 vs. 2, and (D) number of years since last birth <5 vs. ≥20 years on disease-free survival (DFS) of invasive breast cancer without TNM stage 0 by intrinsic subtypes. ^a^I-squared describes the percentage of the variability in effect estimates that is due to heterogeneity rather than sampling error.
(PPTX)

S1 Table Baseline characteristics of remained and excluded subjects because of follow-up loss. (DOCX)

S2 Table The reproductive factors and prognosis of invasive breast cancer patients without TNM stage 0 in Seoul Breast Cancer Study (SEBCS). (DOCX)
