# Supplementary material for: Tumor Subtype-Specific Associations of Hormone-Related Reproductive Factors on Breast Cancer Survival
Source: PLoS One. 2015 Apr 14;10(4):e0123994. doi: 10.1371/journal.pone.0123994 (PMC4397050; doi:10.1371/journal.pone.0123994)
Supplement: S1 File — aThe sample size was not sufficient to analyze the stratified analysis by age categories. (PPTX) [file pone.0123994.s001.pptx]

## Slide 1
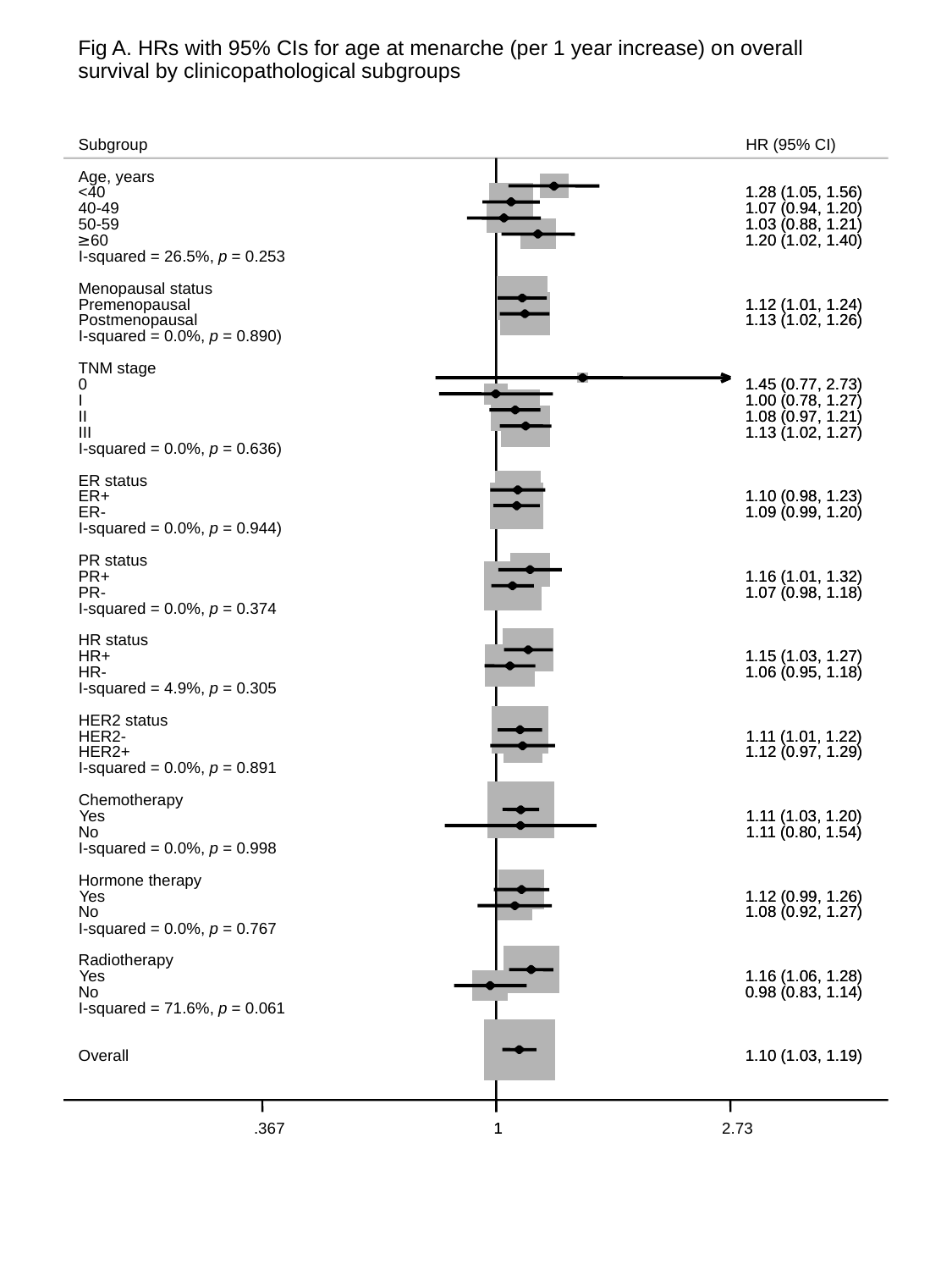

# Fig A. HRs with 95% CIs for age at menarche (per 1 year increase) on overall survival by clinicopathological subgroups
Subgroup
HR (95% CI)
Age, years
<40
1.28 (1.05, 1.56)
1.28 (1.05, 1.56)
40-49
1.07 (0.94, 1.20)
1.07 (0.94, 1.20)
50-59
1.03 (0.88, 1.21)
1.03 (0.88, 1.21)
≥60
1.20 (1.02, 1.40)
1.20 (1.02, 1.40)
I-squared = 26.5%, p = 0.253
Menopausal status
Premenopausal
1.12 (1.01, 1.24)
1.12 (1.01, 1.24)
Postmenopausal
1.13 (1.02, 1.26)
1.13 (1.02, 1.26)
I-squared = 0.0%, p = 0.890)
TNM stage
1.45 (0.77, 2.73)
1.45 (0.77, 2.73)
0
I
1.00 (0.78, 1.27)
1.00 (0.78, 1.27)
II
1.08 (0.97, 1.21)
1.08 (0.97, 1.21)
III
1.13 (1.02, 1.27)
1.13 (1.02, 1.27)
I-squared = 0.0%, p = 0.636)
ER status
ER+
1.10 (0.98, 1.23)
1.10 (0.98, 1.23)
ER-
1.09 (0.99, 1.20)
1.09 (0.99, 1.20)
I-squared = 0.0%, p = 0.944)
PR status
PR+
1.16 (1.01, 1.32)
1.16 (1.01, 1.32)
PR-
1.07 (0.98, 1.18)
1.07 (0.98, 1.18)
I-squared = 0.0%, p = 0.374
HR status
HR+
1.15 (1.03, 1.27)
1.15 (1.03, 1.27)
HR-
1.06 (0.95, 1.18)
1.06 (0.95, 1.18)
I-squared = 4.9%, p = 0.305
HER2 status
HER2-
1.11 (1.01, 1.22)
1.11 (1.01, 1.22)
HER2+
1.12 (0.97, 1.29)
1.12 (0.97, 1.29)
I-squared = 0.0%, p = 0.891
Chemotherapy
Yes
1.11 (1.03, 1.20)
1.11 (1.03, 1.20)
No
1.11 (0.80, 1.54)
1.11 (0.80, 1.54)
I-squared = 0.0%, p = 0.998
Hormone therapy
Yes
1.12 (0.99, 1.26)
1.12 (0.99, 1.26)
No
1.08 (0.92, 1.27)
1.08 (0.92, 1.27)
I-squared = 0.0%, p = 0.767
Radiotherapy
Yes
1.16 (1.06, 1.28)
1.16 (1.06, 1.28)
No
0.98 (0.83, 1.14)
0.98 (0.83, 1.14)
I-squared = 71.6%, p = 0.061
Overall
1.10 (1.03, 1.19)
1.10 (1.03, 1.19)
.367
1
1
2.73

## Slide 2
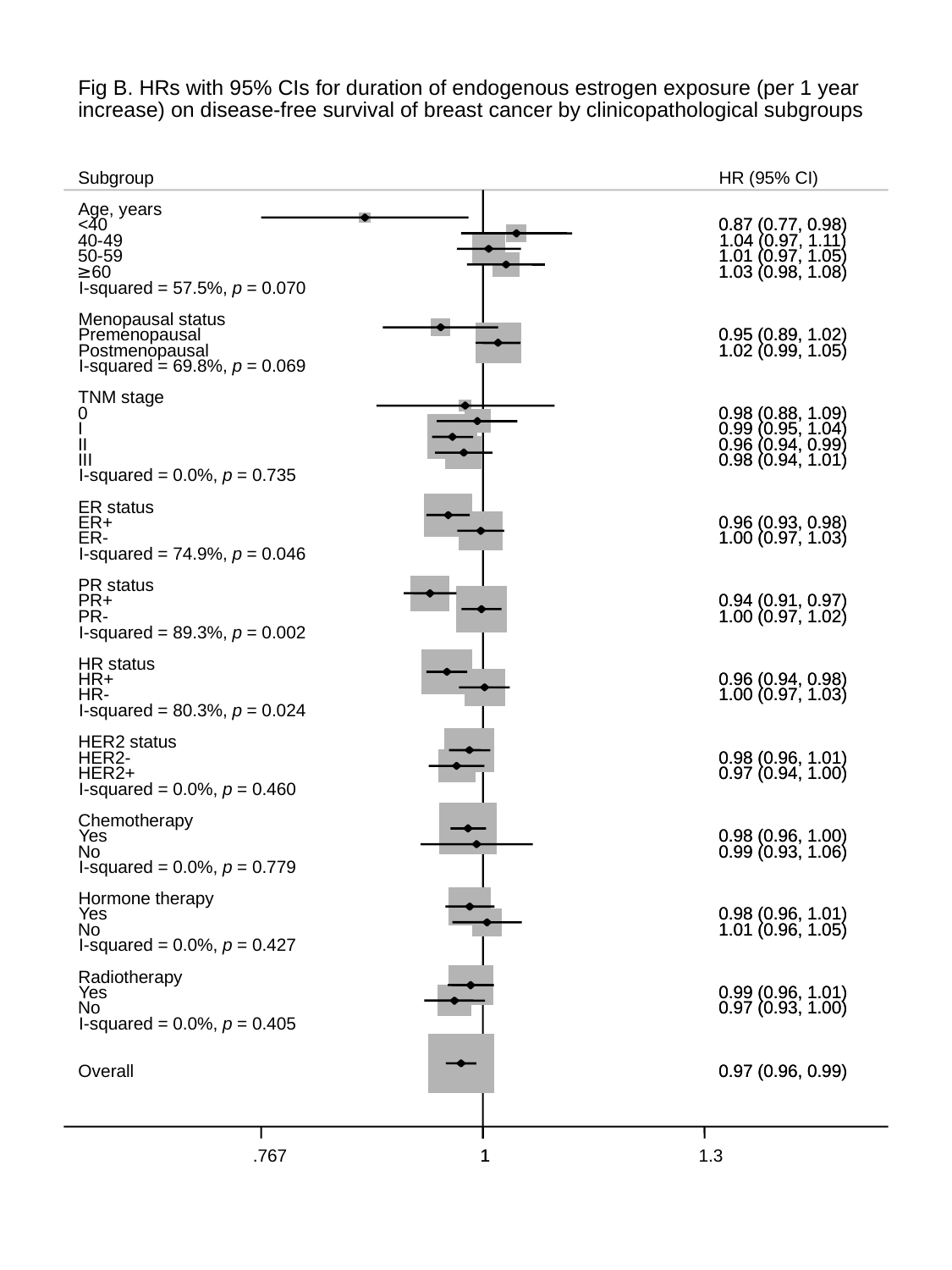

# Fig B. HRs with 95% CIs for duration of endogenous estrogen exposure (per 1 year increase) on disease-free survival of breast cancer by clinicopathological subgroups
Subgroup
HR (95% CI)
Age, years
<40
0.87 (0.77, 0.98)
0.87 (0.77, 0.98)
40-49
1.04 (0.97, 1.11)
1.04 (0.97, 1.11)
1.01 (0.97, 1.05)
1.01 (0.97, 1.05)
50-59
≥60
1.03 (0.98, 1.08)
1.03 (0.98, 1.08)
I-squared = 57.5%, p = 0.070
Menopausal status
Premenopausal
0.95 (0.89, 1.02)
0.95 (0.89, 1.02)
Postmenopausal
1.02 (0.99, 1.05)
1.02 (0.99, 1.05)
I-squared = 69.8%, p = 0.069
TNM stage
0
0.98 (0.88, 1.09)
0.98 (0.88, 1.09)
I
0.99 (0.95, 1.04)
0.99 (0.95, 1.04)
II
0.96 (0.94, 0.99)
0.96 (0.94, 0.99)
III
0.98 (0.94, 1.01)
0.98 (0.94, 1.01)
I-squared = 0.0%, p = 0.735
ER status
ER+
0.96 (0.93, 0.98)
0.96 (0.93, 0.98)
ER-
1.00 (0.97, 1.03)
1.00 (0.97, 1.03)
I-squared = 74.9%, p = 0.046
PR status
PR+
0.94 (0.91, 0.97)
0.94 (0.91, 0.97)
PR-
1.00 (0.97, 1.02)
1.00 (0.97, 1.02)
I-squared = 89.3%, p = 0.002
HR status
HR+
0.96 (0.94, 0.98)
0.96 (0.94, 0.98)
HR-
1.00 (0.97, 1.03)
1.00 (0.97, 1.03)
I-squared = 80.3%, p = 0.024
HER2 status
0.98 (0.96, 1.01)
0.98 (0.96, 1.01)
HER2-
0.97 (0.94, 1.00)
0.97 (0.94, 1.00)
HER2+
I-squared = 0.0%, p = 0.460
Chemotherapy
Yes
0.98 (0.96, 1.00)
0.98 (0.96, 1.00)
No
0.99 (0.93, 1.06)
0.99 (0.93, 1.06)
I-squared = 0.0%, p = 0.779
Hormone therapy
Yes
0.98 (0.96, 1.01)
0.98 (0.96, 1.01)
1.01 (0.96, 1.05)
1.01 (0.96, 1.05)
No
I-squared = 0.0%, p = 0.427
Radiotherapy
0.99 (0.96, 1.01)
0.99 (0.96, 1.01)
Yes
No
0.97 (0.93, 1.00)
0.97 (0.93, 1.00)
I-squared = 0.0%, p = 0.405
Overall
0.97 (0.96, 0.99)
0.97 (0.96, 0.99)
.767
1
1
1.3

## Slide 3
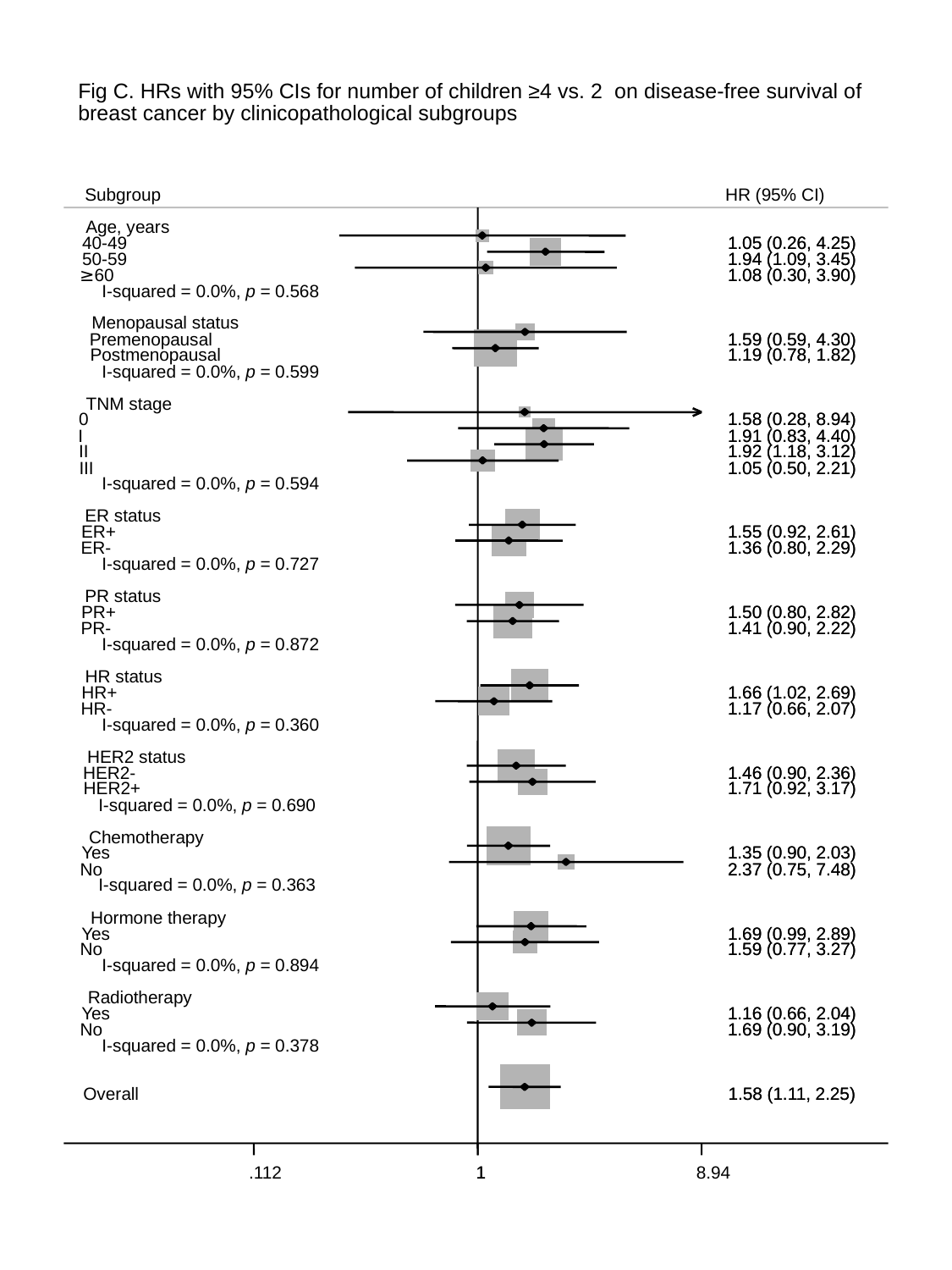

# Fig C. HRs with 95% CIs for number of children ≥4 vs. 2 on disease-free survival of breast cancer by clinicopathological subgroups
Subgroup
HR (95% CI)
Age, years
40-49
1.05 (0.26, 4.25)
1.05 (0.26, 4.25)
50-59
1.94 (1.09, 3.45)
1.94 (1.09, 3.45)
≥60
1.08 (0.30, 3.90)
1.08 (0.30, 3.90)
I-squared = 0.0%, p = 0.568
Menopausal status
Premenopausal
1.59 (0.59, 4.30)
1.59 (0.59, 4.30)
Postmenopausal
1.19 (0.78, 1.82)
1.19 (0.78, 1.82)
I-squared = 0.0%, p = 0.599
TNM stage
0
1.58 (0.28, 8.94)
1.58 (0.28, 8.94)
I
1.91 (0.83, 4.40)
1.91 (0.83, 4.40)
II
1.92 (1.18, 3.12)
1.92 (1.18, 3.12)
III
1.05 (0.50, 2.21)
1.05 (0.50, 2.21)
I-squared = 0.0%, p = 0.594
ER status
ER+
1.55 (0.92, 2.61)
1.55 (0.92, 2.61)
ER-
1.36 (0.80, 2.29)
1.36 (0.80, 2.29)
I-squared = 0.0%, p = 0.727
PR status
PR+
1.50 (0.80, 2.82)
1.50 (0.80, 2.82)
PR-
1.41 (0.90, 2.22)
1.41 (0.90, 2.22)
I-squared = 0.0%, p = 0.872
HR status
HR+
1.66 (1.02, 2.69)
1.66 (1.02, 2.69)
HR-
1.17 (0.66, 2.07)
1.17 (0.66, 2.07)
I-squared = 0.0%, p = 0.360
HER2 status
HER2-
1.46 (0.90, 2.36)
1.46 (0.90, 2.36)
HER2+
1.71 (0.92, 3.17)
1.71 (0.92, 3.17)
I-squared = 0.0%, p = 0.690
Chemotherapy
Yes
1.35 (0.90, 2.03)
1.35 (0.90, 2.03)
No
2.37 (0.75, 7.48)
2.37 (0.75, 7.48)
I-squared = 0.0%, p = 0.363
Hormone therapy
Yes
1.69 (0.99, 2.89)
1.69 (0.99, 2.89)
No
1.59 (0.77, 3.27)
1.59 (0.77, 3.27)
I-squared = 0.0%, p = 0.894
Radiotherapy
Yes
1.16 (0.66, 2.04)
1.16 (0.66, 2.04)
No
1.69 (0.90, 3.19)
1.69 (0.90, 3.19)
I-squared = 0.0%, p = 0.378
Overall
1.58 (1.11, 2.25)
1.58 (1.11, 2.25)
.112
1
1
8.94

## Slide 4
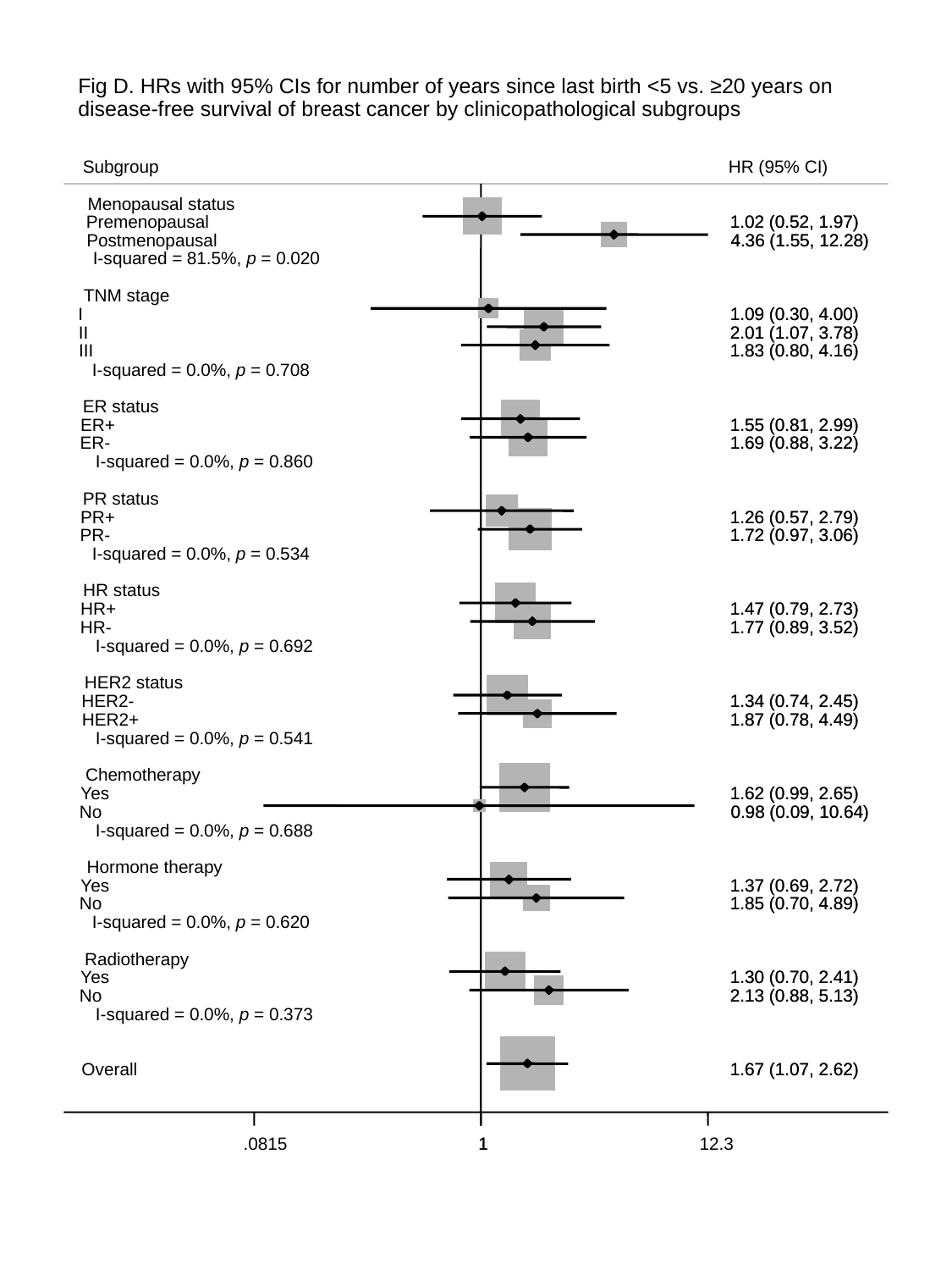

# Fig D. HRs with 95% CIs for number of years since last birth <5 vs. ≥20 years on disease-free survival of breast cancer by clinicopathological subgroups
Subgroup
HR (95% CI)
Menopausal status
Premenopausal
1.02 (0.52, 1.97)
1.02 (0.52, 1.97)
Postmenopausal
4.36 (1.55, 12.28)
4.36 (1.55, 12.28)
I-squared = 81.5%, p = 0.020
TNM stage
I
1.09 (0.30, 4.00)
1.09 (0.30, 4.00)
II
2.01 (1.07, 3.78)
2.01 (1.07, 3.78)
III
1.83 (0.80, 4.16)
1.83 (0.80, 4.16)
I-squared = 0.0%, p = 0.708
ER status
ER+
1.55 (0.81, 2.99)
1.55 (0.81, 2.99)
ER-
1.69 (0.88, 3.22)
1.69 (0.88, 3.22)
I-squared = 0.0%, p = 0.860
PR status
PR+
1.26 (0.57, 2.79)
1.26 (0.57, 2.79)
PR-
1.72 (0.97, 3.06)
1.72 (0.97, 3.06)
I-squared = 0.0%, p = 0.534
HR status
HR+
1.47 (0.79, 2.73)
1.47 (0.79, 2.73)
HR-
1.77 (0.89, 3.52)
1.77 (0.89, 3.52)
I-squared = 0.0%, p = 0.692
HER2 status
1.34 (0.74, 2.45)
1.34 (0.74, 2.45)
HER2-
HER2+
1.87 (0.78, 4.49)
1.87 (0.78, 4.49)
I-squared = 0.0%, p = 0.541
Chemotherapy
1.62 (0.99, 2.65)
1.62 (0.99, 2.65)
Yes
0.98 (0.09, 10.64)
0.98 (0.09, 10.64)
No
I-squared = 0.0%, p = 0.688
Hormone therapy
Yes
1.37 (0.69, 2.72)
1.37 (0.69, 2.72)
1.85 (0.70, 4.89)
1.85 (0.70, 4.89)
No
I-squared = 0.0%, p = 0.620
Radiotherapy
Yes
1.30 (0.70, 2.41)
1.30 (0.70, 2.41)
No
2.13 (0.88, 5.13)
2.13 (0.88, 5.13)
I-squared = 0.0%, p = 0.373
Overall
1.67 (1.07, 2.62)
1.67 (1.07, 2.62)
.0815
1
1
12.3
