# Supplementary material for: Tumor Subtype-Specific Associations of Hormone-Related Reproductive Factors on Breast Cancer Survival
Source: PLoS One. 2015 Apr 14;10(4):e0123994. doi: 10.1371/journal.pone.0123994 (PMC4397050; doi:10.1371/journal.pone.0123994)
Supplement: S2 File — aI-squared describes the percentage of the variability in effect estimates that is due to heterogeneity rather than sampling error. (PPTX) [file pone.0123994.s002.pptx]

## Slide 1
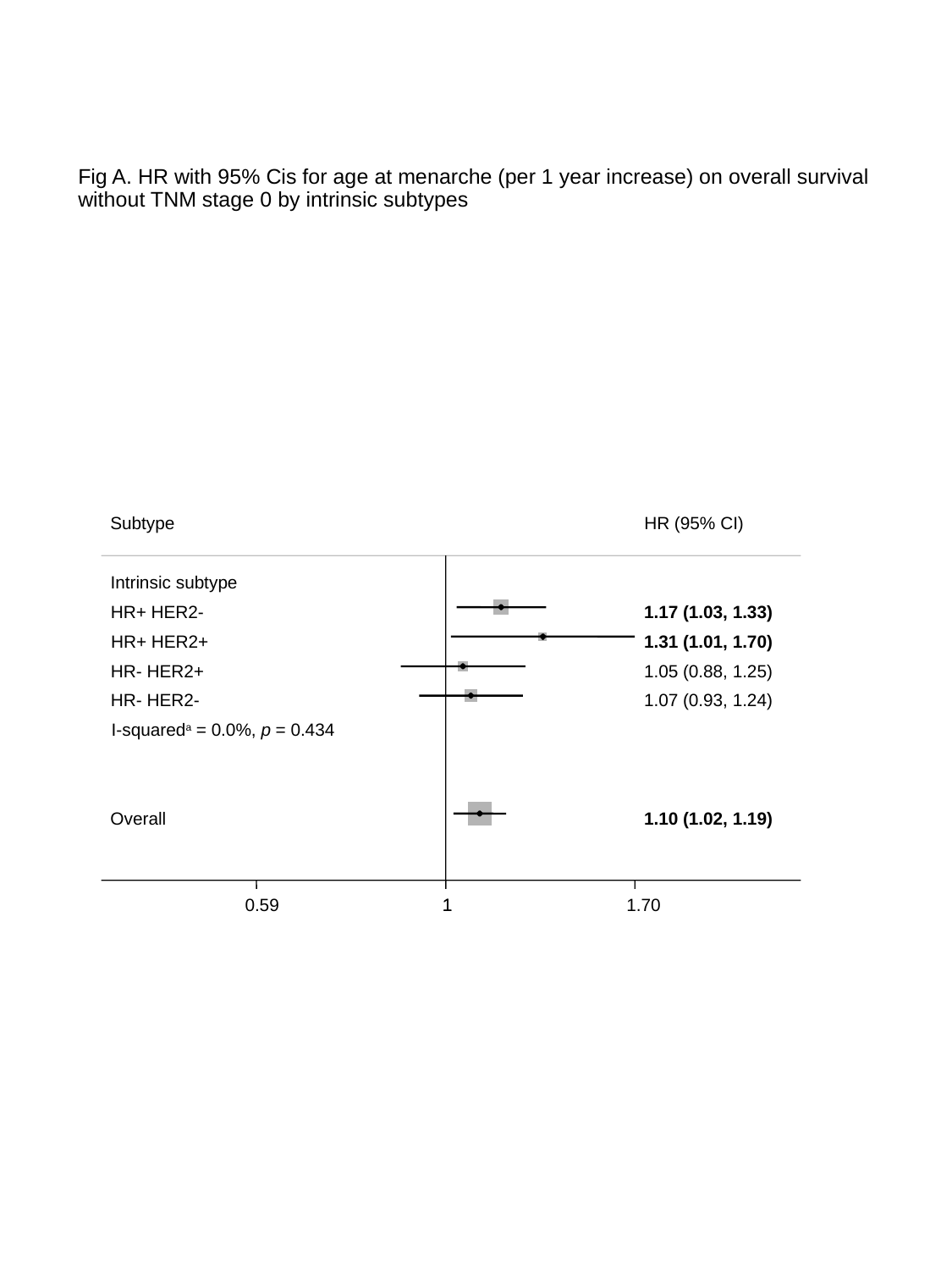

# Fig A. HR with 95% Cis for age at menarche (per 1 year increase) on overall survival without TNM stage 0 by intrinsic subtypes
Subtype
HR (95% CI)
Intrinsic subtype
HR+ HER2-
1.17 (1.03, 1.33)
HR+ HER2+
1.31 (1.01, 1.70)
HR- HER2+
1.05 (0.88, 1.25)
HR- HER2-
1.07 (0.93, 1.24)
I-squareda = 0.0%, p = 0.434
Overall
1.10 (1.02, 1.19)
0.59
1
1
1.70

## Slide 2
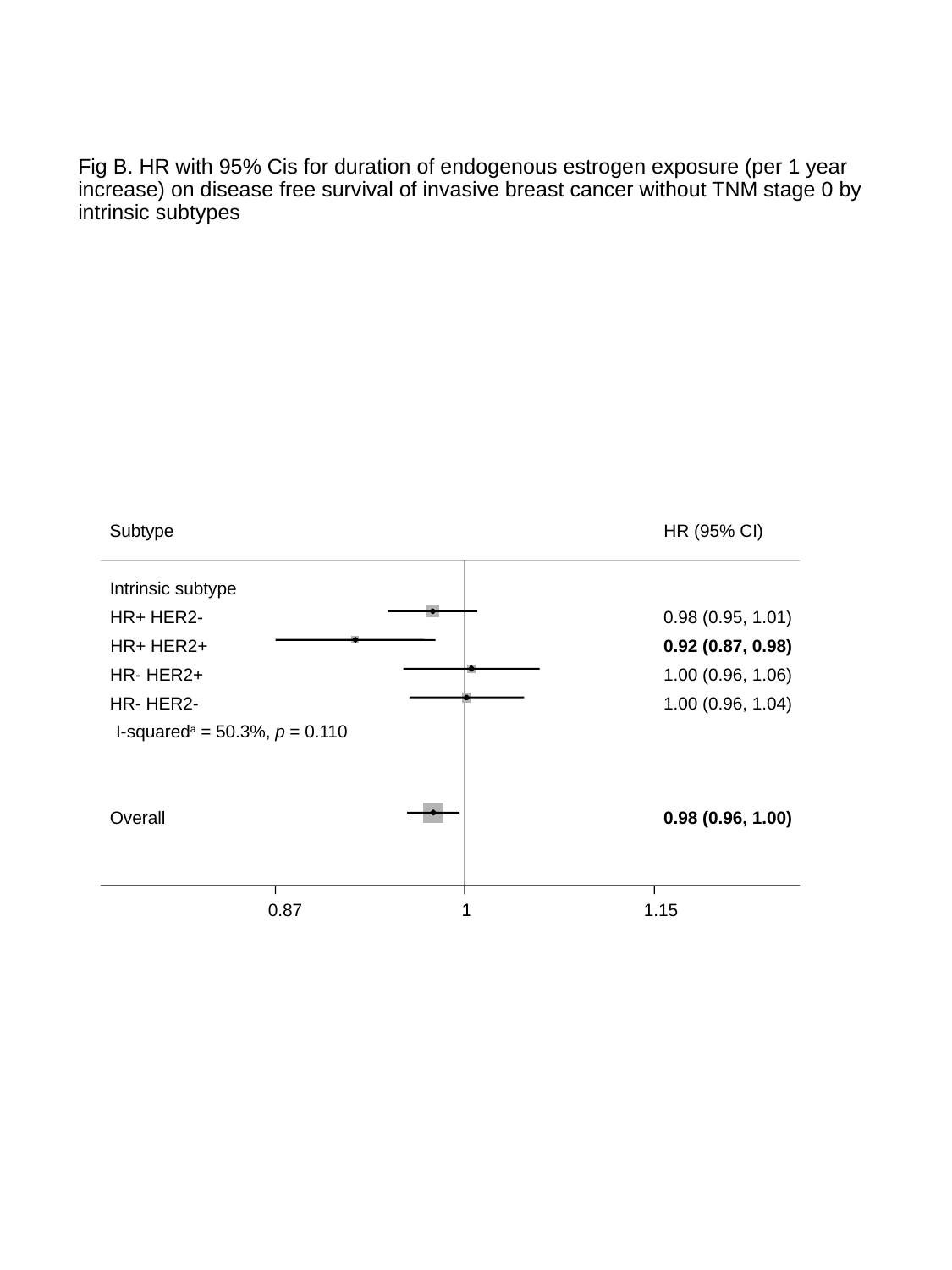

# Fig B. HR with 95% Cis for duration of endogenous estrogen exposure (per 1 year increase) on disease free survival of invasive breast cancer without TNM stage 0 by intrinsic subtypes
Subtype
HR (95% CI)
Intrinsic subtype
HR+ HER2-
0.98 (0.95, 1.01)
HR+ HER2+
0.92 (0.87, 0.98)
HR- HER2+
1.00 (0.96, 1.06)
HR- HER2-
1.00 (0.96, 1.04)
I-squareda = 50.3%, p = 0.110
Overall
0.98 (0.96, 1.00)
0.87
1
1
1.15

## Slide 3
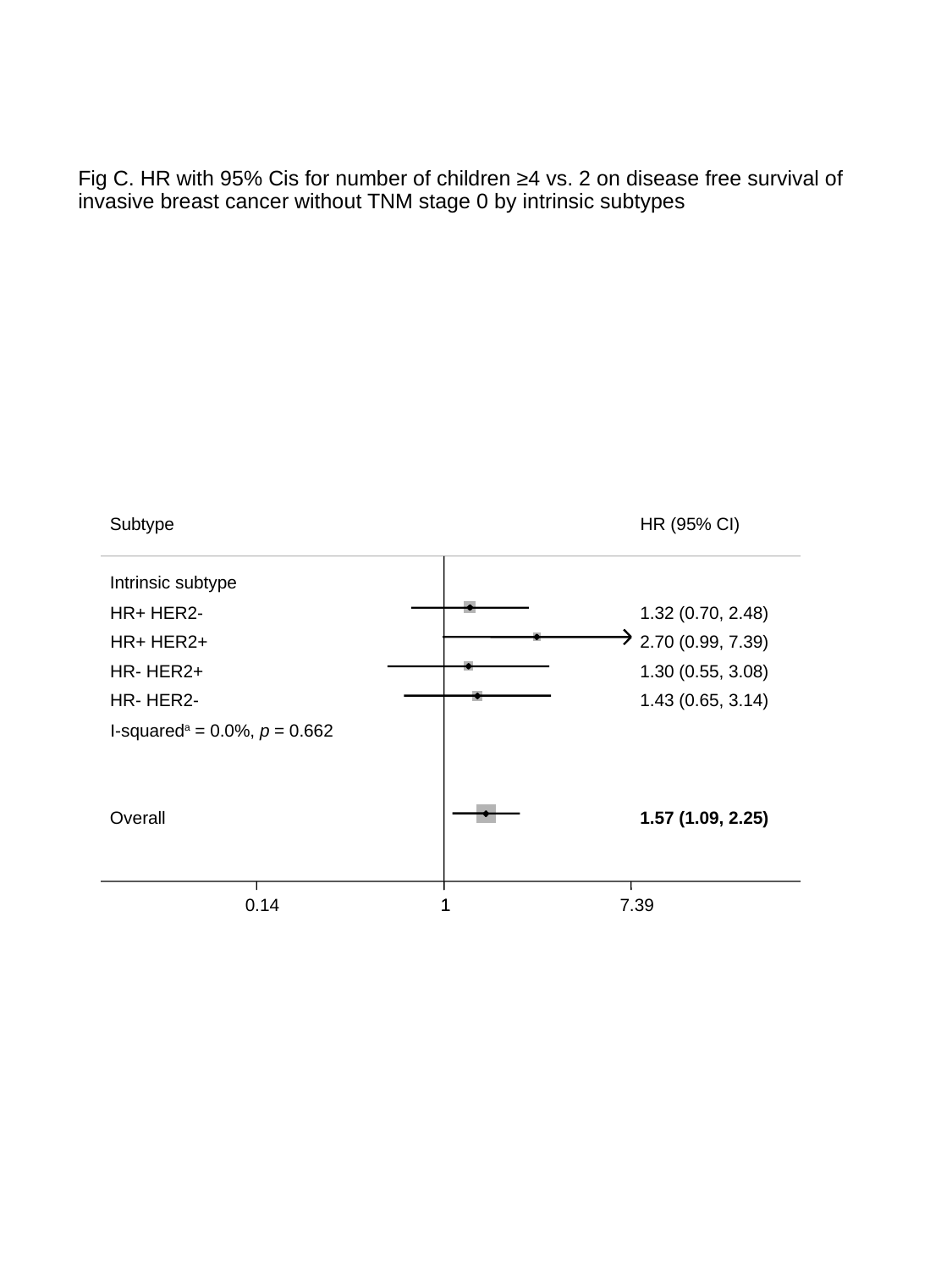

# Fig C. HR with 95% Cis for number of children ≥4 vs. 2 on disease free survival of invasive breast cancer without TNM stage 0 by intrinsic subtypes
Subtype
HR (95% CI)
Intrinsic subtype
HR+ HER2-
1.32 (0.70, 2.48)
HR+ HER2+
2.70 (0.99, 7.39)
HR- HER2+
1.30 (0.55, 3.08)
HR- HER2-
1.43 (0.65, 3.14)
I-squareda = 0.0%, p = 0.662
Overall
1.57 (1.09, 2.25)
0.14
1
1
7.39

## Slide 4
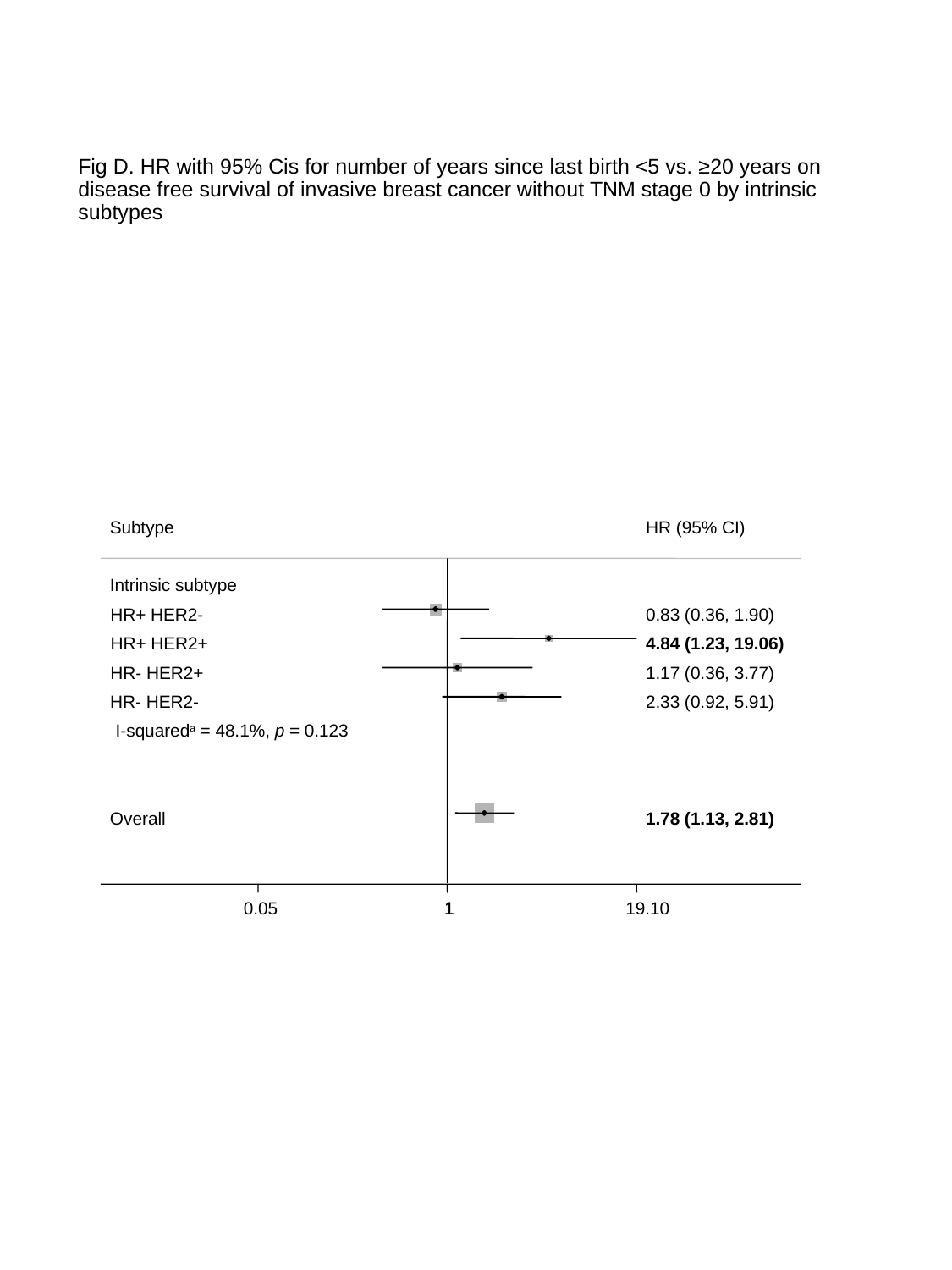

# Fig D. HR with 95% Cis for number of years since last birth <5 vs. ≥20 years on disease free survival of invasive breast cancer without TNM stage 0 by intrinsic subtypes
Subtype
HR (95% CI)
Intrinsic subtype
HR+ HER2-
0.83 (0.36, 1.90)
HR+ HER2+
4.84 (1.23, 19.06)
HR- HER2+
1.17 (0.36, 3.77)
HR- HER2-
2.33 (0.92, 5.91)
I-squareda = 48.1%, p = 0.123
Overall
1.78 (1.13, 2.81)
0.05
1
1
19.10
