# Supplementary material for: Tumor Subtype-Specific Associations of Hormone-Related Reproductive Factors on Breast Cancer Survival
Source: PLoS One. 2015 Apr 14;10(4):e0123994. doi: 10.1371/journal.pone.0123994 (PMC4397050; doi:10.1371/journal.pone.0123994)
Supplement: S1 Table — (DOCX) [file pone.0123994.s003.docx]

| S1 Table. Baseline characteristics of remained and excluded subjects because of follow-up loss. | | | | | | |
| --- | --- | --- | --- | --- | --- | --- |
| Characteristics | Remained subjects in the analysis | | Excluded subjects because of follow-up loss | | | *P*^a^ |
|  | (N=3,430) | | (N=175) | | |  |
|  | N | (%) | | N | (%) |  |
| Age at diagnosis, years |  |  | |  |  | 0.41 |
| <40 | 674 | (19.7) | | 42 | (24.0) |  |
| 40-49 | 1,443 | (42.1) | | 72 | (41.1) |  |
| 50-59 | 871 | (25.4) | | 37 | (21.1) |  |
| ≥60 | 442 | (12.9) | | 24 | (13.7) |  |
| Family history of breast cancer |  |  | |  |  | 0.80 |
| no | 3,266 | (95.2) | | 166 | (94.9) |  |
| yes | 162 | (4.7) | | 9 | (5.1) |  |
| BMI, kg/m^2^ |  |  | |  |  | 0.11 |
| <18.5 | 119 | (3.5) | | 10 | (5.7) |  |
| 18.5-22.9 | 1,675 | (48.8) | | 92 | (52.6) |  |
| 23.0-24.9 | 819 | (23.9) | | 30 | (17.1) |  |
| ≥25.0 | 790 | (23.0) | | 42 | (24.0) |  |
| Menopausal status |  |  | |  |  | 0.54 |
| premenopausal | 2,145 | (62.5) | | 113 | (64.6) |  |
| postmenopausal | 1,259 | (36.7) | | 60 | (34.3) |  |
| TNM stage |  |  | |  |  | 0.47 |
| 0-I | 1,503 | (43.8) | | 64 | (36.6) |  |
| II | 1,441 | (42.0) | | 52 | (29.7) |  |
| III | 485 | (14.1) | | 15 | (8.6) |  |
| unknown | 1 | (0.0) | | 44 | (25.1) |  |
| Intrinsic subtypes |  |  | |  |  | 0.21 |
| HR+ HER2- | 1,615 | (47.1) | | 63 | (36.0) |  |
| HR+ HER2- | 398 | (11.6) | | 8 | (4.6) |  |
| HR- HER2+ | 395 | (11.5) | | 13 | (7.4) |  |
| HR- HER2- | 473 | (13.8) | | 22 | (12.6) |  |
| unknown | 549 | (16.0) | | 69 | (39.4) |  |
| ^a^Chi-square test. | | | | | | |
