# Supplementary material for: Tumor Subtype-Specific Associations of Hormone-Related Reproductive Factors on Breast Cancer Survival
Source: PLoS One. 2015 Apr 14;10(4):e0123994. doi: 10.1371/journal.pone.0123994 (PMC4397050; doi:10.1371/journal.pone.0123994)
Supplement: S2 Table — (DOCX) [file pone.0123994.s004.docx]

| S2 Table. The reproductive factors and prognosis of invasive breast cancer patients without TNM stage 0 in Seoul Breast Cancer Study (SEBCS) | | | | | | | | | | |
| --- | --- | --- | --- | --- | --- | --- | --- | --- | --- | --- |
| Characteristics | All | | Deaths | | HR^a^ | (95% CI) | Recurrences | | HR^a^ | (95% CI) |
|  | (N=3,073) | | (N=264, 8.6%) | |  |  | (N=508, 16.5%) | |  |  |
|  | N | (%) | N | (%) |  |  | N | (%) |  |  |
| Age at menarche, years | |  |  |  |  |  |  |  |  |  |
| ≤13 | 634 | (20.6) | 39 | (14.8) | 0.80 | (0.56-1.16) | 91 | (17.9) | 0.89 | (0.69-1.13) |
| 14-15 | 1,433 | (46.6) | 121 | (45.8) | 1.00 | ref. | 240 | (47.2) | 1.00 | ref. |
| ≥16 | 970 | (31.6) | 100 | (37.9) | 1.23 | (0.92-1.63) | 170 | (33.5) | 0.98 | (0.79-1.20) |
| per 1 year increase | |  |  |  | 1.10 | (1.02-1.19) |  |  | 1.02 | (0.97-1.08) |
| Age at menopause among postmenopausal women, years | | | | | | | | | | |
| ≤47 | 325 | (28.4) | 21 | (20.2) | 0.97 | (0.56-1.69) | 47 | (22.1) | 0.88 | (0.60-1.28) |
| 47-51 | 400 | (35.0) | 35 | (33.7) | 1.00 | ref. | 71 | (33.3) | 1.00 | ref. |
| ≥52 | 361 | (31.6) | 43 | (41.4) | 1.28 | (0.80-2.03) | 81 | (38.0) | 1.14 | (0.82-1.57) |
| per 1 year increase | |  |  |  | 1.02 | (0.98-1.07) |  |  | 1.03 | (1.00-1.05) |
| Duration of endogenous estrogen exposure, years | | | | | | | | | | |
| ≤27 | 970 | (31.6) | 95 | (36.0) | 1.00 | ref. | 172 | (33.9) | 1.00 | ref. |
| 28-33 | 1,081 | (35.2) | 85 | (32.2) | 0.82 | (0.58-1.17) | 168 | (33.1) | 0.92 | (0.71-1.18) |
| ≥34 | 922 | (30.0) | 73 | (27.7) | 0.78 | (0.51-1.20) | 146 | (28.7) | 0.89 | (0.66-1.21) |
| per 1 year increase | |  |  |  | 0.96 | (0.93-0.98) |  |  | 0.98 | (0.96-1.00) |
| Duration of endogenous estrogen exposure before first full-term pregnancy (FFTP), years | | | | | | | | | | |
| ≤9 | 1,008 | (32.8) | 98 | (37.1) | 1.00 | ref. | 187 | (36.8) | 1.00 | ref. |
| 10-13 | 1,087 | (35.4) | 87 | (33.0) | 0.76 | (0.56-1.03) | 163 | (32.1) | 0.82 | (0.66-1.02) |
| ≥14 | 928 | (30.2) | 72 | (27.3) | 0.80 | (0.58-1.12) | 148 | (29.1) | 0.94 | (0.74-1.19) |
| per 1 year increase | |  |  |  | 0.98 | (0.95-1.00) |  |  | 0.99 | (0.98-1.01) |
| Menstrual cycle |  |  |  |  |  |  |  |  |  |  |
| regular | 2,052 | (66.8) | 194 | (73.5) | 1.00 | ref. | 350 | (68.9) | 1.00 | ref. |
| irregular | 440 | (14.3) | 53 | (20.1) | 0.99 | (0.70-1.40) | 91 | (17.9) | 0.93 | (0.72-1.20) |
| Parity |  |  |  |  |  |  |  |  |  |  |
| parous | 2,817 | (91.7) | 236 | (89.4) | 1.00 | ref. | 462 | (90.9) | 1.00 | ref. |
| nulliparous | 256 | (8.3) | 28 | (10.6) | 1.45 | (0.97-2.18) | 46 | (9.1) | 1.24 | (0.91-1.70) |
| Age at first full-term pregnancy (FFTP) among parous women, years | | | | | | | | | | |
| ≤24 | 969 | (34.4) | 81 | (34.3) | 1.00 | ref. | 169 | (36.6) | 1.00 | ref. |
| 25-27 | 1,027 | (36.5) | 87 | (36.9) | 1.01 | (0.74-1.38) | 157 | (34.0) | 0.91 | (0.73-1.14) |
| ≥28 | 804 | (28.5) | 67 | (28.4) | 1.03 | (0.74-1.45) | 134 | (29.0) | 1.06 | (0.84-1.34) |
| per 1 year increase | |  |  |  | 0.99 | (0.95-1.03) |  |  | 1.00 | (0.97-1.03) |
| Number of children among parous women | | | | | | | | | | |
| 1 | 440 | (15.6) | 44 | (18.6) | 1.40 | (0.98-1.99) | 74 | (16.0) | 1.15 | (0.88-1.49) |
| 2 | 1,636 | (58.1) | 120 | (50.9) | 1.00 | ref. | 239 | (51.7) | 1.00 | ref. |
| 3 | 481 | (17.1) | 45 | (19.1) | 1.19 | (0.83-1.70) | 92 | (19.9) | 1.22 | (0.95-1.57) |
| ≥4 | 209 | (7.4) | 25 | (10.6) | 1.57 | (0.94-2.62) | 50 | (10.8) | 1.57 | (1.09-2.25) |
| Age at last birth among parous women, years | | | | | | | | | | |
| ≤28 | 875 | (31.1) | 90 | (38.1) | 1.00 | ref. | 151 | (32.7) | 1.00 | ref. |
| 29-31 | 757 | (26.9) | 61 | (25.9) | 0.77 | (0.56-1.08) | 130 | (28.1) | 1.01 | (0.80-1.28) |
| ≥32 | 803 | (28.5) | 78 | (33.1) | 1.00 | (0.73-1.36) | 138 | (29.9) | 1.07 | (0.84-1.35) |
| per 1 year increase | |  |  |  | 1.00 | (0.97-1.03) |  |  | 1.00 | (0.98-1.03) |
| Time since last birth among parous women, years | | | | | | | | | | |
| ≥20 | 1,106 | (39.3) | 112 | (47.5) | 1.00 | ref. | 209 | (45.2) | 1.00 | ref. |
| 15-19 | 416 | (14.8) | 30 | (12.7) | 0.92 | (0.57-1.48) | 54 | (11.7) | 0.81 | (0.57-1.14) |
| 10-14 | 391 | (13.9) | 28 | (11.9) | 0.92 | (0.54-1.57) | 48 | (10.4) | 0.78 | (0.53-1.15) |
| 5-9 | 322 | (11.4) | 28 | (11.9) | 1.16 | (0.65-2.05) | 55 | (11.9) | 1.12 | (0.74-1.68) |
| <5 | 208 | (7.4) | 32 | (13.6) | 2.02 | (1.08-3.77) | 54 | (11.7) | 1.78 | (1.13-2.82) |
| per 1 year decrease | |  |  |  | 1.00 | (0.97-1.03) |  |  | 1.00 | (0.98-1.03) |
| Duration of breastfeeding among parous women^b^, months | | | | | | | | | | |
| never | 580 | (20.6) | 37 | (15.7) | 1.00 | ref. | 88 | (19.1) | 1.00 | ref. |
| ≤12 | 780 | (27.7) | 74 | (31.4) | 1.23 | (0.81-1.86) | 118 | (25.5) | 0.89 | (0.67-1.19) |
| 13-24 | 539 | (19.1) | 53 | (22.5) | 1.24 | (0.80-1.93) | 90 | (19.5) | 0.94 | (0.69-1.28) |
| >24 | 566 | (20.1) | 68 | (28.8) | 1.65 | (1.00-2.71) | 123 | (26.6) | 1.16 | (0.81-1.65) |
| per 1 month increase | |  |  |  | 1.01 | (1.00-1.01) |  |  | 1.01 | (1.00-1.01) |
| Abbreviations: Overall survival (OS); disease-free survival (DFS); hazard ratio (HR); confidence interval (CI). | | | | | | | | | | |
| ^a^Adjusted for age, recruiting centers, menopausal status, TNM stage, and intrinsic subtypes. | | | | | | | | | | |
| ^b^Additionally adjusted for number of children. | | | | | | | | | | |
